# Farm costs and benefits of antimicrobial use reduction on broiler farms in Dar es Salaam, Tanzania

**DOI:** 10.3389/frabi.2022.1011929

**Published:** 2022-11-15

**Authors:** Rogers R. Azabo, Janeth I. George, Stephen E. Mshana, Mecky I. Matee, Sharadhuli I. Kimera

**Affiliations:** ^1^ Department of Veterinary Microbiology, Parasitology and Biotechnology, College of Veterinary Medicine and Biomedical Sciences, Sokoine University of Agriculture, Morogoro, Tanzania; ^2^ Livestock Program, National Livestock Resources Research Institute, Kampala, Uganda; ^3^ Southern African Centre for Infectious Disease Surveillance (SACIDS) Foundation for One Health, Sokoine University of Agriculture, Morogoro, Tanzania; ^4^ Department of Veterinary Medicine and Public Health, College of Veterinary Medicine and Biomedical Sciences, Sokoine University of Agriculture, Morogoro, Tanzania; ^5^ Department of Microbiology and Immunology, Weill Bugando School of Medicine, Catholic University of Health and Allied Sciences, Mwanza, Tanzania; ^6^ Department of Microbiology and Immunology, School of Medicine, Muhimbili University of Health and Allied Sciences, Dar es Salaam, Tanzania

**Keywords:** antimicrobial use reduction, broiler, costs and benefits, Dar es salaam, mclnerney model, Tanzania

## Abstract

Of all animal derived-food, the demand for poultry meat is the most dynamic. The poultry sector can meet this demand only by introducing intensive production where antimicrobial use is inevitable. Bacterial infection prevention and control is an important factor in intensive livestock production. Antibiotics are an effective and relatively inexpensive means of preventing and controlling infections, thus maintaining animal health and productivity. The aim of this study was to gain insight into the costs and benefits of various scenarios of antimicrobial use reduction at broiler farms in Dar es Salaam, Tanzania. This study focused on the economic impact of an average broiler farm. Costs and benefits for various scenarios of antimicrobial use reduction levels were projected by a partial budget framework using the Mclnerney model. The disease cost of the current situation was US$225. On reduction of antimicrobial use by 20% the avoidable disease cost was US$ 31, by 50% was US$ 83 and by 100% was US$ 147. A reduction in antibiotic use can only be achieved if better alternatives are available to combat disease. In conclusion, the model predicts that reducing antibiotic use increases production costs. Future studies on antimicrobial use reduction’s impact on morbidity and mortality and the efficiency of additional control and other measures of producing poultry meat without high concentrations of antibiotics are necessary.

## 1 Introduction

The poultry sector plays an important role in the agricultural sector not only in Tanzania but worldwide (EFSA, 2015). Tanzania’s poultry production system is grouped into three: indigenous/traditional, improved chicken and specialized commercial chicken systems (Da Silva et al., 2017). The indigenous/traditional system is the most predominant; and dominated by indigenous chicken, which is raised mainly on extensive scavenging and its purpose is twofold. It is used for both meat and egg production. At maturity, they weigh 1.5 kg with low levels of egg production of about 50/year (Da Silva et al., 2017). They provide most of the poultry meat and eggs consumed in rural areas and approximately 20% in urban areas (Msami, 2008). The commercial specialized chicken system. This production system is intensive and involves both layer and broiler chicken. At maturity, the chicken’s live weight is between 1.5 -2 kg with a high egg production of 270/year. It is mostly practiced in urban and peri-urban areas and accounts for more than 80% of meat and egg consumption in those areas (Msami, 2008). According to the National Bureau of Statistics of Tanzania, 2019/20 (National Sample Census of Agriculture) the poultry population is estimated at 87.6 million birds which include 43.7 million indigenous chickens, 18.5 million layers and 12.8 million broilers (TNBS, 2021). Over the years, small-scale commercial systems have emerged raising broilers and layers (FAO, 2019). Tanzania like any other low-middle Income Country (LMIC), poultry (chicken) production is on the rise due to the increased demand for animal dietary protein intake (Nonga et al., 2009) to meet the needs of the growing population. This has led to intensive poultry production where antimicrobial use (AMU) is inevitable (Van Boeckel et al., 2019). In poultry production, antimicrobials are generally administered through water or feed to the entire flock purposely for treatment, disease prevention and growth promotion (Page and Gautier, 2012; Poole and Sheffield, 2013). However, in all large poultry-producing countries (Nataliya et al., 2019) antimicrobial usage is permitted for disease prevention.

Poultry production, especially broiler production provides an ordinary form of cheap protein source (Mahama et al., 2013) and is among the intensive livestock production systems (Hughes et al., 2008). Turnover rate is very high, and the rate of return on investment is the highest among livestock enterprises (Gbigbi, 2017). In addition, broiler meat is nutritious. Tanzania produced 80,601.3 metric tonnes of poultry meat in the financial year 2019/2020 as recorded in the formal market (MLF, 2020). One of the prerequisites for beneficial livestock production is good animal health. Morbid animals cause production loss through increased death, reduced feed conversion, and stunted growth. In broiler production, throughout the growing period, mortality is about 5% (Scanes et al., 2004). A high mortality rate indicates a problem. In Tanzania, broiler production has a short production cycle of 4-6 weeks (Msami, 2008).

Broiler farms have the highest proportion of resistant bacterial strains compared to other livestock species and increased annual antibiotic dosages per chicken. Therefore, it makes sense to reduce antibiotic use on broiler farms. AMU on farms is essential for the maintenance of animal health and productivity (Robinson et al., 2016). However, the widespread use of antibiotics in livestock has become a global concern due to increased resistance, threatening treatment options in both veterinary and human medicine (Bywater, 2004; Prescott, 2008; Apata, 2009). These antibiotic-resistant bacteria may infect humans through contact with animals, food animal products, or *via* the environment (Marshall and Levy, 2011; Da Costa et al., 2013). Due to increased death and associated treatment costs, human antimicrobial resistance (AMR) has shifted from being a medical problem to a socio-economical problem, prompting policymakers to focus on mitigation strategies in humans by containing AMU in animal health (WHO/FAO/OIE, 2016). A policy to reduce AMU must be effective in achieving the goal of reducing AMR risk while keeping the economic consequences on livestock farms at acceptable levels (Erik, 2011). Tang et al. (2017) reported that evidence exists that shows antibiotic usage in animal husbandry contributes to a surprising increase in human treatment failures, although the quantitative contribution to this failure is unclear (Lhermie et al., 2018). This uncertainty justifies policies to reduce AMU in food animals.

In addition to treatment goals, economic goals are also achieved by AMU in livestock production, since the occurrence of disease threatens the efficiency and profitability of the production process on the farms (Lhermie et al., 2017). Decreases in AMU can result in increased mortality and/or morbidity in diseased livestock, increase disease incidence within the herd, and as a result, reduce products entering the food chain (Lhermie et al., 2018).

Several studies have been conducted, and some are ongoing in Tanzania on the effects of antibiotic-resistant bacteria on human health (Erik, 2011) of animal origin which mainly focuses on microbiology (Nonga and Muhairwa, 2010; Rugumisa et al., 2016; Katakweba et al., 2018; Subbiah et al., 2020; Kiiti et al., 2021; Mgaya et al., 2021; Kimera et al., 2021). However, there is dearth of information on the economic impact of reducing antimicrobial use in livestock production in Tanzania. To address this knowledge gap, a study was conducted to gain insight into the costs and benefits of three different hypothetical scenarios of antimicrobial reduction (20%,50%.100%) in broiler production in Tanzania by comparing them to the baseline scenario which is the current field practices of AMU. The economic effects (costs and benefits) of the hypothetical scenarios were determined by a partial budgeting model.

## 2 Materials and methods

### 2.1 Study design, area and farm recruitment

**Figure 1:**
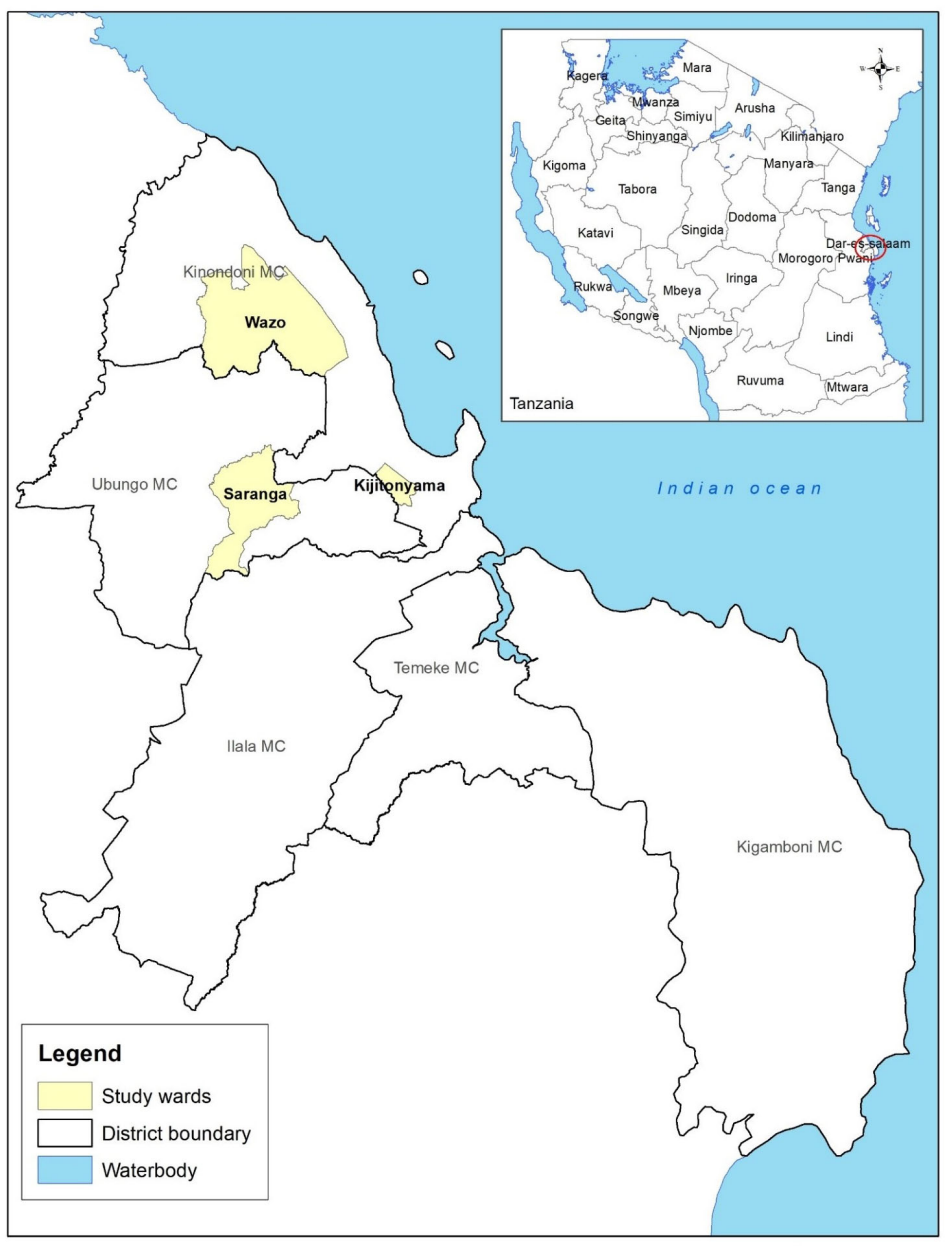
Map of study districts (wards) in Dar es Salaam, Tanzania.

A cross-sectional study was conducted between February and March 2021, on farms raising broilers (chickens) in Kinondoni and Ubungo districts that form part of Dar es Salaam city, Tanzania. These areas were purposively chosen because of the numerous broiler farming activities. Three wards were included namely, Wazo, Kijitonyama and Saranga (**Figure 1**). A list of 55 Broiler farms that had been in production continuously for at least one year was provided by the livestock officers of the study wards. Broiler (chicken) farms were selected based on the number of birds (≥100) with properly maintained records. A simple random sampling was conducted and only 22 broiler farms with nearly all the parameters to be used in the model were identified.

### 2.2 Data collection

A pre-tested questionnaire (**Supplementary Table 1**) was used to capture the data required to complete the model for the estimation of the economic effects (costs and benefits) on the reduction of antimicrobial use described previously by McInerney (1996). The following information related to the flock was obtained: (i) the number of day-old chicks (DOCs) bought and dead on the farm (mortality) during a specific time frame (weekly and cumulative percentages); (ii) Any observed clinical signs; signs of respiratory infections (coughing, wheezing, sneezing and nasal discharge), enteric infection (diarrhea), and locomotive signs like lameness, all these clinical signs were considered under morbidity (iii) Use of health-supporting products like vitamins. Other information obtained was: feed costs, the purchase price of DOCs, weight at slaughter, age at marketing, feed consumption, cost of antimicrobials, and total cost per broiler (live and processed). Respondents were anonymized at the time of data collection. Farmers were asked to retain all used containers/bottles/packages of antimicrobial products used on their flocks. The researcher visited the farms on four occasions during the production cycle which lasted between 4 – 6 weeks to cross check the data. The first visit was on the restocking day, where the DOC was weighed, the information required for recording was compiled and the provision of posters with visual images of the common clinical signs in flocks to farmers. Data on flock-related variables were collected at later visits during the production cycle.

### 2.3 Data analysis

**Figure 2:**
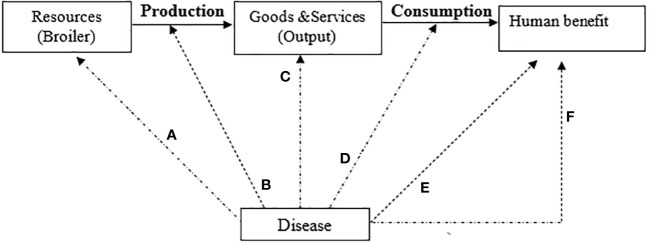
Diseases in the livestock production system (McInerney Model, 1996).

This study used a model (**Figure 2**) developed by McInerney (1996) to analyze the costs and benefits of antimicrobial reduction on broiler farms in Dar es Salaam. This model is based on biological and economic parameters (Lhermie et al., 2018) of disease effects on livestock and projected costs and benefits using a partial budget framework. We assumed some parameters which are suitable for this geographical location. A simulation model was used to determine some of the parameters used in this model (Mclnerney). The model quantifies the economic effects of reducing antimicrobial use at the farm level and has a one-year time horizon. The costs have been expressed in United States dollars for a better and easy understanding of the calculations using the dollar rate as of 2021.

### 2.4 The model

The model consists of three main parts; to calculate (i) mortality and morbidity losses from potential bacterial infections, (ii) costs of management changes to reduce risks due to bacterial infections, and (iii) net costs of various scenarios of antibiotic reduction based on costs of antibiotic treatment.

Livestock diseases in a given production system reduce the efficiency with which resources are transformed into products. Negative impacts can occur directly or indirectly. Direct and indirect losses can occur as described by the model of Mclnerney in **Figure 2**.

Direct effects:

(a) Destruction of basic resources (mortality of productive animals);
(b) Reduction or lowering of the efficiency of the production process (morbidity)
(c) Lowered quality or quantity of output of the production process

Indirect effects:

(d) Losses due to additional costs to compensate for the reduced growth due to morbidity.
(e) Affecting the well-being of humans (through zoonoses such as ESBLs producing *E. coli*;
(f) A broader range of economic impacts that reduce societal value derived from livestock production (restricted trade in animal products, reduced consumer awareness of food safety).

### 2.5 Antimicrobial usage reduction scenarios

#### 2.5.1 Baseline/current scenario

Under this scenario, we assume that antimicrobials are used in broiler production without the implementation of restrictive measures.

**Scenario A**. There are three levels of antimicrobial reduction (20%, 50% and 100%). In this scenario, reducing antimicrobial use is assumed to increase the risk of health problems. It also assumes that the farmer has not taken steps to prevent or control the infection.

**Scenario B**. In this three-step antimicrobial reduction (20%, 50%, 100%) scenario, it is assumed that farmers will invest in infection control strategies to maintain the health of their flocks. In addition, farmers are expected to change their technical management to make up for lost production. As antimicrobial reductions decrease, the number and types of interventions farmers invest in increase. The underlying assumption is that the less antibiotics are used, the higher the cost of avoidable diseases, so as antibiotic use decreases, farmers invest more in interventions. antibiotics increases. It is assumed that the change in management brings the risk to the current level for all three stages in this scenario.

**Figure 3:**
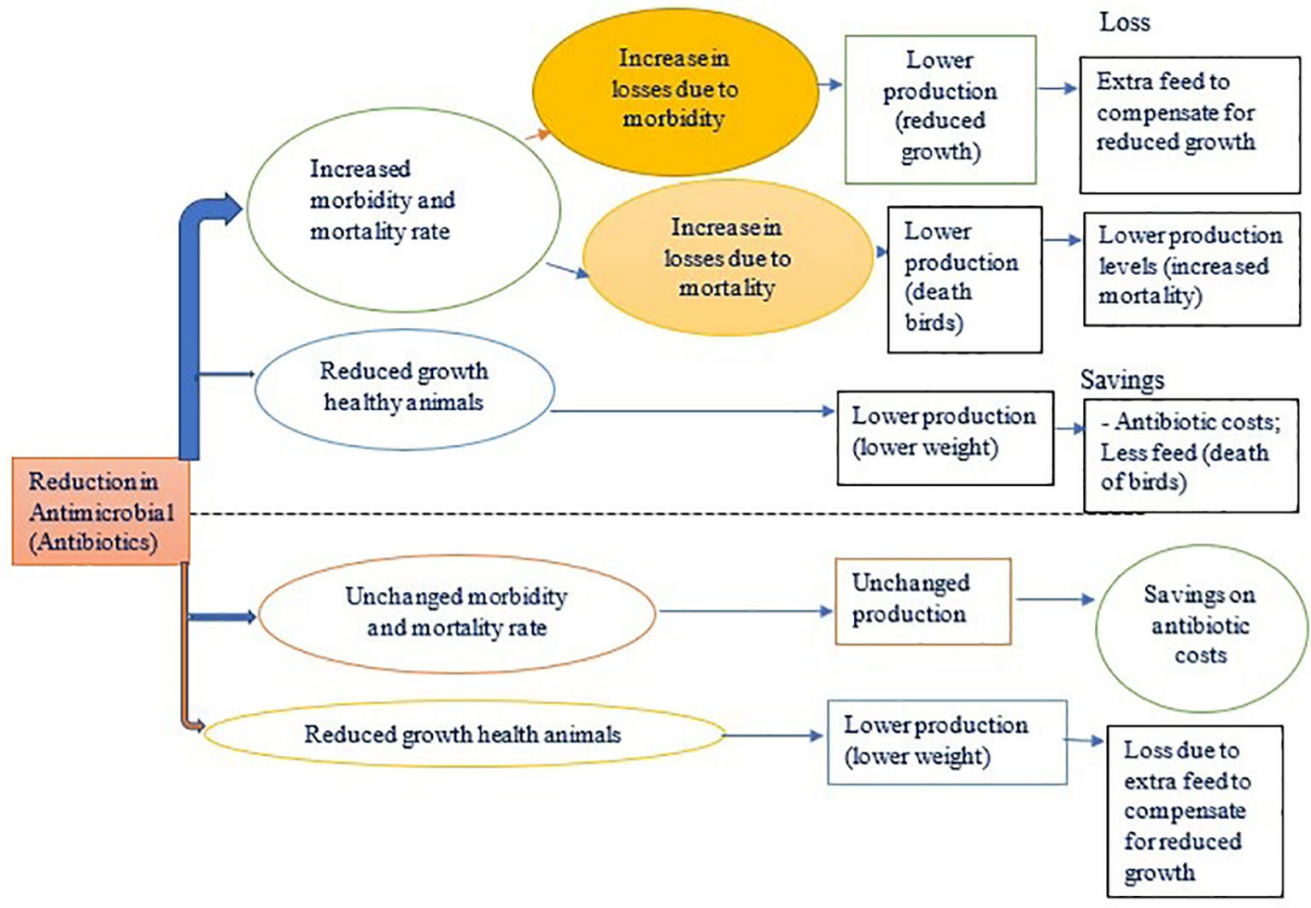
Possible effects of antimicrobial use reduction on production.

The reduction of antibiotic usage may have two effects: i) an increase in the severity of the bacterial infection, and ii) minimal impact on animal health (**Figure 3**). As the frequency and severity of bacterial infections increase, farmers can look at the prevention and control of bacterial infection by investing in extra control measures such as a new drinking system.

The inputs used in the model are shown in **Tables 1A**–**C** below. Some inputs are based on calculations. The calculations are given in the model description below

**Table 1A:** Technological and economic data model inputs.

Input	Description	Value	Unit	Source
N_area_	Stocking density	24	chick/m^2^	assumption
N_rounds_	Number of rounds per year	6		Farm data
N_total_	Total number of chickens per farm	36000	#chickens/yr/farm	calculations
P_doc_	Purchase price (day old chick)	0.76	$	Farm data
W	Weight of chicken	1.5	Kg	Farm data
ΔFCR	Increase in feed conversion	0.05		assumption
FCR	Feed conversion ratio	0.04	Kg/day	calculations
ΔFCRA	Decrease in feed conversion rate due to antibiotic reduction	0.016		assumption
% W	Relative reduction in weight	0.05		assumption
P_f_	Price of feed (weighed average)	0.50	US $	calculations
P_m_	Price of meat	3.06	US $	Farm data
P_vitamin_	Price of vitamins per chicken per treatment	0.01	US$/animal	assumption
C_t_	Cost of antibiotic treatment per chicken/year	0.18	US$/animal	calculations
P_probiotics_	Price of probiotics per chicken per treatment	0.01	US $/animal	assumption
CD	Destruction costs	0.02	US$/animal	calculations
C_f_	Cost of the increased FCR	0.04	US$/animal	calculations
C_rw_	Cost of reduced weight	0.25	US$/animal	calculations
RC_ventilation_	Replacement cost ventilation system	15.00	US$/m^2^	Farm records
RC_drink_	Replacement cost drinking system	11.90	US$/m^2^	Farm data
RC_cooling_	Investment in a heating & cooling system	13.00	US$ m^2^	Farm records
LS	Lifespan of investment	12.50	Years	Farm records
L_i_	Effect on labour cost	0.0		assumption
λ _i_	The scale factor of investment i	1.0		assumption

**Table 1B:** Mortality rates, current situation and antimicrobial reduction levels.

	Parameter	Current	20% antibiotic reduction	50% antibiotic reduction	100% antibiotic reduction
MRT_digestion_	Mortality rate due to digestion problem	0.0100	0.0120	0.0140	0.0160
MRT_respiratory_	Mortality rate due to respiratory problem	0.0040	0.0044	0.0054	0.0064
MRT_locomotion_	Mortality rate due to locomotion problem	0.0040	0.0044	0.0054	0.0064
MRT_first week_	Mortality rate of first-week problem	0.0140	0.0154	0.0182	0.0210

**Table 1C:** Morbidity rates, current situation and antimicrobial reduction levels.

	Parameter	Current	20% antibiotic reduction	50% antibiotic reduction	100% antibiotic reduction
MBR_digestion_	Morbidity rate due to digestion problem	0.28	0.32	0.38	0.48
MBR _respiratory_	Morbidity rate due to respiratory problem	0.12	0.14	0.17	0.22
MBR _locomotion_	Morbidity rate due to locomotion problem	0.33	0.39	0.48	0.63
MBR _first week_	Morbidity rate of first-week problem	0.12	0.14	0.17	0.22

### 2.6 Calculations

The calculation methods used in the current study are those used by Erik, 2011 as described below.

#### 2.6.1 Calculations of broiler chicken loss due to mortality

In the model, the number of broiler death is considered a loss due to mortality.


(1) LMR=MRT×N×TVM+CD

LMR = Loss due to mortality; MRT = Mortality rate; N= number of chickens on the farm per year; TVM = total value of chicken at the time of mortality.

N = number of chickens per round multiplied by7 (assumed number of rounds per year).

In this model, growing chickens represent a value used to quantify the financial loss due to mortality

TVM = Purchase price + cumulative feed costs + overhead costs and profit margin. The total value of chicken at time mortality is calculated for each week, **Table 2**.

**Table 2:** The total value of a broiler chicken at different ages during its life.

Bird age in weeks	1	2	3	4	5	6
Chick purchase price	0.76	0.76	0.76	0.76	0.76	0.76
Overhead costs	0.07	0.14	0.20	0.26	0.33	0.39
Profit margin	0.58	0.58	0.58	0.58	0.58	0.58
Cumulative feed costs	0.08	0.16	0.33	0.47	0.61	0.72
Total (US $)	1.49	1.64	1.87	2.07	2.28	2.45

In the model, losses due to mortality following the death of animals as a result of disease outbreaks are considered for a specific disease.

#### 2.6.2 Calculation of broiler chicken loss due to morbidity

Morbidity negatively affects animal growth. This model assumes an increase in feed conversion ratio (FCR) as a result of infection. FCR is a measure of how efficiently feed mass (kg) is converted to body weight by the animal. This ratio is obtained by dividing the consumed feed by the weight gained.


(2) $${\text{C}}_{\text{f}}=\text{W}\times \Delta \text{FCR}\times \text{Pf}$$

Whereby C**_f_ =** Cost of the increased feed conversion ratio (FCR); W= chicken weight, ΔFCR= increase in FCR, P_f =_ feed price

In the model, it is assumed that chickens will lose weight at the end of the growing period (42days) due to infection and increased FCR.

Loss of the lower weight (C_rw_)


(3) $$\text{Crw}=\%\text{W}\times \text{W}\times {\text{P}}_{\text{m}}$$

Whereby % W = relative weight reduction, W= weight of normal chicken, P_m_ = meat price

Total loss due to morbidity (LMB) = Eqs (2 + 3) x number of chickens that suffers from the disease

Number of diseased chickens = Morbidity rate (MBR) x infected number of birds. The infected number of bird is determined by multiplying the entire flock number on the farm (N) by1 and subtract the mortality rate (MRT).


(4) $$\text{LMB}=\text{MBR}\times \left(\text{N}\times (1-\text{MRT})\right)\times ({\text{C}}_{\text{f}}+\text{Crw})$$

This model assumes that morbidity increases as antibiotic use decreases. In the three hypothetical scenarios, morbidity is assumed to increase at the same rate as antibiotics decrease.

#### 2.6.3 Calculation of losses due to stunted growth of healthy flocks

Reduction of antimicrobial use reduces the growth rate of healthy flocks. This model assumes that chickens receive additional amounts of feed to compensate for their reduced growth.


(5) $$\text{CRG}=\text{W}+\Delta \text{FCRA}\times {\text{P}}_{\text{f}}\times \left(\text{N}(1-\text{MRT})\right)$$

Whereby CRG = Cost of reduced growth, W = chicken weight, ΔFCRA = the decrease in FCR, P_f_ = feed price, (N (1- MRT) = total sum of animals corrected for mortality

In the model, avoidable disease cost is defined as the total cost of disease consisting of mortality and morbidity losses.

Avoidable cost of categorical disease = (Loss in mortality + Loss in morbidity) x probability of infection occurring.

Avoidable costs of each category of infection are calculated separately and each scenario of antimicrobial reduction.

### 2.7 Cost due to management changes

The model hypothesizes that reducing antibiotic use increases the severity of the disease. Farmers can alternate animal health practices to minimize the likelihood of disease outbreaks in their flocks. Alteration of animal health cares in the model is a means to prevent or control infection.

On reduction of antimicrobial use a farmer will invest in preventive or control measures; for instance, improvement in drinking system and additional cleaning during and before production rounds.

Investment cost (INV) = Replacement cost (RC) x Unit (m^2^). The replacement cost is divided by its lifespan (LS).

Farm size affects investment costs, and economies of scale apply to some investments. In this case this is taken into account by the scaling factor λ. Investment in new equipment affects the amount of time farmers spend on the farm (Erik, 2011). This has positive effects on operations, such as increased monitoring time, and negative effects, such as reduced operations due to computer use. The impact on labor costs is represented by L (Erik, 2011).


(6) $$\text{INV}=\frac{\text{RC}\times \text{unit}}{\text{LS}}-\text{λ}+\text{L}$$

Investment costs in animal health management such as drinking water systems is part of the model.

The replacement cost (RC) for the investments included in the model is based on price per square meter and assumed lifetime of 12.5 years

A square meter of the farm = number of birds (chicken) restocked per round (shed capacity)/an assumed occupancy of 24 animals per square meter (Erik, 2011).

Technical management, in the model makes up for production losses due to reduced growth (morbidity). This model assumes that farmers will risk whatever it takes to make up for losses with technical management changes, such as adding vitamins.

The assumption is a reduction of antimicrobial use will lead to improvement in technical management.


(7) CTM=P×unit

CTM = Cost of technical measure; P = Price per unit (animal kg or treatments) x number of units used per year (Erik, 2011).

At different levels of antimicrobial use reduction, variations exist in the level of technical and animal health management. The assumption is that the higher the level of reduction in antimicrobial use, the more the management levels will be applied.

### 2.8 Savings on reduced antibiotic use

Reduction of antimicrobial use, reduces the cost of antimicrobials and other costs associated with antimicrobial treatment.


(8) $$\text{Ca}=\%\text{AR}\times {\text{C}}_{\text{t}}\times \text{N}$$

C_a_ **=** Cost on reduction, % AR **=** Relative reduction in antimicrobial use, Ct = Cost of treating one chicken per year, N = number of chickens (Eq 5).

## 3 Results based on the model

### 3.1 Economic impact on average poultry (broiler) farm

Costs and benefits of three levels of antibiotic reduction (20%, 50%, 100%) are calculated for an average broiler farm by the model (Erik, 2011). For this study, an average broiler farm is a farm with a floor area of ​​250 m^2^ and a restocking capacity of 6,000 chickens each round.

The model assumes that there are 7 production rounds per year, so the flock number on the farm is 36,000 per year.

### 3.2 Disease costs

In this model, the cost of the disease is represented by loss due to morbidity and mortality of chicken. This is calculated separately for each specific condition (first week, locomotory, respiratory, digestive issues). **Table 3** shows the current situation and the calculated cost of disease for lower antibiotic levels.

**Table 3:** Disease cost calculated values for the current situation and the three levels of antimicrobial use reduction.

Parameter	Current costs	Costs 20% antimicrobial reduction	Costs 50% antimicrobial reduction	Costs 100% antimicrobial reduction
Digestion problems (US $)	72	84	99	117
Respiratory problems (US$)	30	33	41	50
Locomotion problems (US$)	51	59	73	93
First week problems (US$)	72	80	95	112
Total (US $)	225	256	308	372
Avoidable disease costs (US$)		31	83	147
Δ% compared to current		14%	37%	65%

In the model, the cost of the disease for the present situation for an average broiler farm is US $ 225. With the reduction of antimicrobial use by 20%, the avoidable disease cost is US $ 31. When reduced by 50% it is US $ 83 while by 100% it is US $ 147. Furthermore, the model estimates the disease cost to increase by 14% when antimicrobial use is reduced by 20% and with 50% and 100% reduction, the disease cost will increase by 37% and 65% respectively.

### 3.3 Additional costs due to changes in management

By reducing the use of antimicrobials, farmers are likely to invest in disease preventive or control measures. The model has two majors. Changes in health care and technical management. This model envisions measures applied to individual stages of antibiotic reduction. In practice, the farmer’s decision depends on the cost-benefit analysis of the measures applied.

Because the impact of extra measures on mortality and morbidity of three level antibiotic reduction is not clear, a number of additional measures are assumed. **Table 3.1** shows the annual costs of different types of measures involved in the scenario. The annual cost calculation assumes a useful life of 12.5 years. Since the period of the model is one year, the annual investment amount is not adjusted for inflation. Total investment in added animal health measures is shown in **Table 3.2**.

**Table 3.1:** Annual cost calculated for additional animal health and technical measures.

	Scenario B		
	20% AB reduction	50% AB reduction	100% AB reduction
Animal health management (INV)			
Investment in the new drinking water system	0	238	238
Investment new ventilation system	0	0	300
Investment in the heating system	260	260	260
Subtotal (US $)	260	498	798
Technical management (CTM)			
Costs increased vitamin intake	0	0	360
Costs of probiotic use	0	360	360
Subtotal (US $)	0	360	720
Total (US$)	260	858	1,518

**Table 3.2:** Calculated total investment for additional animal health measures.

	Scenario B		
	20% AB reduction	50% AB reduction	100% AB reduction
Investment in the new drinking water system	0	2975	2975
Investment new ventilation system	0	0	3750
Investment in the heating & cooling system	3250	3250	3250
Total (US $)	3250	6225	9,975

This model assumes that farmers invest in heating and cooling systems when antimicrobial use is reduced by 20%. The annual cost of this investment is 260 USD and the total investment is 3250 USD. With a 50% reduction in antimicrobial content, farmers are expected to invest in new drinking water systems, new heating and cooling systems and probiotics. The total annual cost of all these actions is $858. For the 50% reduction scenario, the total investment in animal health measures is $6,225. With a 100% discount, the farmer invests in every measure of the model. In this scenario, the annual investment cost is $1,518 and the total investment in additional animal care is $9,975.

### 3.4 Savings due to reduced antimicrobial use

Antimicrobial use reduction results in reduced cost of treatment as shown in **Table 3.3**.

**Table 3.3:** Calculated savings on reduced antimicrobial use.

	20% AB reduction	50% AB reduction	100% AB reduction
Savings due to reduced antimicrobial use (US$)	1,296	3,240	6,480

The annual cost savings from reducing antibiotic use by 20% is $1,296. A 50% reduction would reduce his treatment costs by $3,240, and a 100% reduction in antibiotic use would be $6,480. The economic savings from reducing antibiotic use are greater than the increased costs of additional management and avoidable disease.

### 3.5 Loss due to reduced growth in healthy animals

Prophylactic use of antimicrobial agents in animals has a positive effect on their growth. This effect is lost when antimicrobial use is reduced. Antibiotic reduction in is believed to lead to a reduction in feed conversion rate. In addition, farmers will make up for reduced growth with additional feed. Feed additions are calculated using the FCR. The cost of these additional amounts of feed is shown in **Table 3.4**.

**Table 3.4:** Loss calculation due to reduced growth of healthy animals.

	20% AB reduction	50% AB reduction	100% AB reduction
Loss due to reduced growth of healthy animals	$ 85	$ 213	$ 425

A 100% reduction in antibiotic use costs US$425 in reduced growth. The model assumed that the 20% loss and 50% reduction scenarios for antibiotics would be 20% and 50% loss for the 100% reduction in antibiotic use.

### 3.6 Economic effects at farm level

Cost and benefits of a reduction in antimicrobial use

The net cost of the scenario is calculated by subtracting the cost of avoidable disease, the cost of additional control measures, and the cost associated with reduced growth of healthy animals due to antibiotic savings.

The changes in the net cost are shown in **Table 3.5**. This figure shows the most significant costs are cost of avoidable disease and the cost of additional control measures. The model assumes that morbidity and mortality will remain at current levels if additional control measures are applied (Scenario B). The same pattern can be seen if we compare the increase in the cost of disease in scenario A with the increase in the cost of additional control measures in scenario B. The net cost increase for scenarios A and B is similar. Therefore, under the assumptions of this model, there is no significant difference in annual net costs between no additional controls (Scenario A) and investing in additional controls (Scenario B).

**Table 3.5:** Annual calculated costs and benefits of antimicrobial use reduction.

Scenario	Avoidable disease costs	Savings antimicrobial use	Additional management	Loss growth	Net costs
Current	225	0	0	0	-225
Scenario A 20% AB reduction	341	1296	0	85	870
Scenario A 50% AB reduction	521	3240	0	213	2506
Scenario A 100% AB reduction	797	6480	0	425	5258
Scenario B 20% AB reduction	225	1296	260	85	726
Scenario B 50% AB reduction	225	3240	858	213	1944
Scenario B 100% AB reduction	225	6480	1518	425	4312

## 4 Discussion

In this study, a model was used to estimate the farm-level economic impact of reducing antimicrobial use in broiler farms. This model calculated the net cost of reducing antibiotics on an average farm.

### 4.1 Costs of disease

This study shows that the use of antibiotics is a relatively inexpensive means to reduce the risk of disease compared to alternatives, such as additional control measures. The results should be interpreted with caution as antimicrobials are still necessary. This model predicts that reducing antibiotic use will increase mortality and morbidity in chickens, thereby increasing the cost of disease. The model estimates that a 20% reduction in antibiotic use would increase total health costs by 14% ($31) for an average farm (6,000 chickens per rotation). A 50% reduction in use increases health care costs by 37% ($83), and a 100% reduction in antibiotic use increases health care costs by 65% ​​($147). This is consistent with a study by Erik (2011) reported in the Netherlands and found that the cost of disease on the average farm (90,000 chickens per rotation) decreased as antibiotic use decreased. increased by 16%, 42%, and 81%20, respectively. %, 50% or 100%. The increase in health care costs can probably be explained by the increase in morbidity. This pattern can be explained by assuming that morbidity increases when antibiotic use decreases at the same rate.

### 4.2 Additional control measures

Additional control measures cost more as compared to antibiotic costs. In Scenario B, farmers take more precautionary measures to prevent and control the disease. The greater the reduction in antibiotics, the more farmers are expected to take additional steps. In practice, the farmers decide which additional control measures to apply based on cost-benefit analyses. Since the benefits of additional control measures are unclear, future studies should focus on this issue. It is believed that additional measures will keep avoidable disease costs at current levels. A farmer is considered to invest in a new heating and cooling system if antibiotic use is reduced by 20%. The annual cost is $260 and the total investment he has is $3,250. If antimicrobial levels drop by 50%, farmers are considered to have invested in new drinking water systems, new heating and cooling systems, and probiotics. The total annual cost of all these measures is US$858, with a total investment of US$6,225. If antimicrobial use were reduced by 100%, farmers would be expected to invest in new ventilation systems and provide additional vitamins, in addition to the measures in the 50% reduction scenario. The annual cost of these measures is US$1,518. Total investment in additional animal health measures (new drinking water, heating and cooling and ventilation systems) for the 100% reduction scenario is USD 9,975. The cost of additional measures is high, as it may require complete replacement of the entire system. However, if the system is in place, the cost of additional actions is minimal.

### 4.3 Savings due to reduced antimicrobial usage

Reducing the use of antibiotics reduces the cost of antibiotic treatment. The savings that can be achieved by reducing antibiotic use are relatively high. A 20% reduction in antibiotic use would reduce disease costs by US$1,296. Reducing antibiotic use by 50% and 100%, respectively, reduces costs by $3,240 and $6,480, respectively. The model does not take into account veterinary visit costs or other costs associated with antibiotic treatment.

### 4.4 Costs and benefits of reducing antimicrobial use

According to this model, reducing antimicrobial usage increases production costs. However, the savings from reducing antibiotic use are higher to cater for the increased costs. Farmers therefore have economic incentives on reduction of antibiotic use and in addition less risk to antibiotic resistance exposure. Loss of production increases when antibiotic treatment is ineffective. The decrease in antibiotic use is also consistent with the switch to sustainable production. At therapeutic level, antibiotic use improves animal health and its welfare. Therefore, reducing antibiotic use can only be achieved if better alternatives to combat disease exists. Future studies should emphasis on the development of these alternatives and measure the effectiveness of these interventions. The model is built on reductionism and therefore designed to compute the cost of reducing antibiotic use. The impact of antibiotic reduction in Tanzania is currently unknown, as there are currently no policies to implement antibiotic reduction.

Knowledge on this topic will assist in making political decisions about whether and to what extent reductions in antibiotic use in animal husbandry are necessary and justified. It should be noted that one of the reasons for reducing the use of antibiotics in animal husbandry is the alleged negative impact of high antibiotic use in animal husbandry on the success of antibiotic treatment in humans.

The results of this study can be used for comparison with studies conducted elsewhere on whether a further increase in the cost, as a result of the antibiotic reduction, will decrease net farm results even more and thus have a considerable negative effect on the income of the farmer.

This study predicts the net cost impact of reducing antibiotic use. The model makes assumptions on the impact of reduced antibiotic usage on mortality and morbidity. Calculating avoidable disease costs is an important component of the model. Future studies should emphasize on measurement of antibiotic reduction impact on the health of the broiler chicken. As this data becomes available, the model can be revised to provide accurate estimates. One of the limitations of this model is the inability to calculate the net cost of a particular antibiotic, rather than the total cost of antibiotics, as shown in this study. This study should be considered as the first of its kind in Tanzania to estimate the cost of reducing antibiotic use on broiler farms. The effect of the reduction of antibiotic use on broiler health is not known. However, this study is without limitations. First, few broiler farms had records that were up to date and thus were not representative of the farms in the study area. Secondly, there are no similar studies done within the country or region which could be compared with the current one to determine whether the additional management measures were high.

## 5 Conclusion

According to this model, reducing antibiotic use increases production costs. Future studies on the effect of antibiotic use reduction on mortality and morbidity is needed. Furthermore, studies on the development and effectiveness of additional intervention measures and other approaches of producing poultry meat without the use of high concentrations of antibiotics is needed

## Data availability statement

The original contributions presented in the study are included in the article/**Supplementary Material**. Further inquiries can be directed to the corresponding author.

## Ethics statement

The ethical clearance was provided by the Medical Research Coordinating Committee of the National Institute of Medical Research of Tanzania (NIMR), (Ref #.NIMR/HQ/R.8a/Vol.IX/3233), and the ethical committee of Sokoine University of Agriculture (Ref# DPRTC/186/3). Written informed consent was obtained from the owners for the participation of their animals in this study.

## Author contributions

Conceptualization, RA. Methodology, JG and RA. Validation, SK and RA. Formal analysis, RA and JG. Investigation, RA. Data curation, RA. Writing original draft preparation, RA. Writing review and editing, RA, JG, SK, MM, and SM. Visualization, all authors. Supervision, SK, MM, and SM. All authors contributed to the article and approved the submitted version.

## Funding

This study was funded by the Government of the United Republic of Tanzania and the World Bank [WB-ACE II Grant PAD1436, IDA [Credit 5799-TZ] through SACIDS Foundation for One Health, Sokoine University of Agriculture, Morogoro, Tanzania.

## Acknowledgments

The authors wish to thank the Government of the United Republic of Tanzania and the World Bank through SACIDS Foundation for One Health for providing a scholarship grant to RA. We appreciate the technical assistance provided by the local government authorities and the SACIDS staff during data collection and the poultry farmers who unconditionally accepted to participate in the study.

## Conflict of interest

The authors declare that the research was conducted in the absence of any commercial or financial relationships that could be construed as a potential conflict of interest.

## Publisher’s note

All claims expressed in this article are solely those of the authors and do not necessarily represent those of their affiliated organizations, or those of the publisher, the editors and the reviewers. Any product that may be evaluated in this article, or claim that may be made by its manufacturer, is not guaranteed or endorsed by the publisher.
