# Supplementary material for: Farm costs and benefits of antimicrobial use reduction on broiler farms in Dar es Salaam, Tanzania
Source: Front Antibiot. 2022 Nov 15;1:1011929. doi: 10.3389/frabi.2022.1011929 (PMC11732011; doi:10.3389/frabi.2022.1011929)
Supplement: Supplementary file 1 [file Table_1.docx]

**Supplementary Table 1: Structured Questionnaire on in-puts for Mclnerney Model for economic modeling on broiler farm production**

Date_______________________________Interviewer________________________________

District______________Ward_______________Interviewee_______________Sex______

**Restocking**

1. How many chicks do you bring per batch?

2. How many batches do you bring in a year?

3 Where do they buy the chicks and at what price?

**Health Status of broilers on the farm**

4. Have you ever experienced any of these risks in your flock?

a) Respiratory b) digestion c) locomotion d) 1st week problem

5. At what age did the bird experience these risks?

a) Week 1 b) Week 2 c) Week 3 d) Week 4 e) Week 5 f) Week 6 g) Week 7

6) Who treats the bird when sick?

a) Veterinarian b) Self Neighbour

b. Which antibiotics did you use, what volumes and for how many days?

**7.** Did you witness any death in your flock due to any of the risk problems mentioned

above? a) Respiratory b) digestion c) Locomotion d) 1st week problem

**Health costs**

8. What was the cost of the antibiotics you used for the treatment of any of the

above conditions?

**Health management**

9. In the event of the above risks did you invest in any of the following?

a) New drinking water system b) New ventilation system c) Floor cooling and heating

d) None of the above

10. If so, how much did it cost?

**Technical management**

11. Apart from antibiotics did you use any other veterinary drug?

a) Yes b) No

12. Which one and how much did it cost?

13. What was it intended for and how long did you use it?

14. Did you provide the birds with any supplements?

a) Yes b) No

**Feeds**

15. If yes for how long and how much does it cost?

16. On the introduction of day-old chicks on the farm, which type of feed do you provide to

them? a) Pellets b) Mash c) Mixture of mash & pellets

16.b) From which manufacturer and why?

17. How many bags do you buy and for how long do you feed them?

18. What is the cost of each bag?

19. When do start feeding them on either growers’ pellet/mash?

20. How many bags do you feed them on and for how long?

21. What is the cost of each bag?

22. When do you introduce the birds to finishers’ pellets/ mash and how many bags?

23. What is the cost of each bag?

24. At what age do you dispose of the birds?

25. By the time you dispose of them how much do they weigh?

Thank you for the kind responses

**For further information, contact:**

**E-mail:** [rogersazabo@gmail.com](mailto:rogersazabo@gmail.com); **Mobile Tel no:** +255 654 641 180
